# Exploring perspectives of stigma and discrimination among people with lived experience of mental health conditions: a co-produced qualitative study

**DOI:** 10.1016/j.eclinm.2024.102509

**Published:** 2024-02-28

**Authors:** Petra C. Gronholm, Sarah Kline, Muskan Lamba, Heidi Lempp, Akerke Mahkmud, Guadalupe Morales Cano, Kriti Vashisht, Norha Vera San Juan, Charlene Sunkel

**Affiliations:** a Centre for Global Mental Health, Institute of Psychiatry, Psychology and Neuroscience, King's College London, London, UK; b United for Global Mental Health, London, UK; c Global Mental Health Peer Network, Southeast Asia, Delhi, India; d Centre for Rheumatic Disease, Department of Inflammation Biology, School of Immunology and Microbial Sciences, Faculty of Life Sciences & Medicine, King's College London, London, UK; e Fundación Mundo Bipolar, Madrid, Spain; f Universidad Jaume I, Valencia, Spain; g Global Mental Health Peer Network, America's Region, TX, USA; h Institute for Global Health, University College London, London, UK; i Rapid Research Evaluation and Appraisal Lab (RREAL), University College London, London, UK; j Global Mental Health Peer Network, Paarl, South Africa

**Keywords:** Mental health-related stigma, Discrimination, Public stigma, Co-production, Qualitative survey

## Abstract

**Background:**

Efforts to understand the mechanisms and consequences of mental health-related stigma and discrimination need to center the perspectives of people affected by these negative impacts, through research efforts that are led or co-led by people with lived experience (PWLE) of mental health conditions.

**Methods:**

This study used co-production principles to explore global perspectives of stigma and discrimination among people meeting the inclusion criteria of identifying as PWLEs and being willing to share their experiences of stigma and discrimination resulting from a diagnosis of a mental health condition, and who had also participated in anti-stigma activities. Participants were recruited online via a self-selecting snowball sampling method. Qualitative data were collected from respondents via an anonymous global online survey conducted between 12/01/2021 and 02/28/2022. The main outcomes assessed were open-ended, qualitative responses to questions exploring experiences of stigma and discrimination, experiences regarding diagnoses, language/terminology related to mental health, impact of stigma and discrimination, and involvement with anti-stigma interventions. Data were synthesised through digital text network analysis and thematic content analysis.

**Findings:**

A total of 198 respondents from over 30 countries across Europe, the Americas, Africa, Asia, and Australia/Oceania were included in the study. The results reflected five themes: 1) the role of language and words; 2) the role of media in perpetuating and reducing stigma; 3) societal reactions to mental health conditions and strategies to cope with these; 4) knowledge about activities to reduce stigma and discrimination and their impact; and 5) personal involvement in activities to reduce stigma and discrimination.

**Interpretation:**

The findings highlight that people with mental health conditions are aware of and experience stigma and discrimination across core domains of daily life. The importance of recognising the key role PWLEs can play in efforts to reduce stigma and discrimination was highlighted, and how they can be appropriately supported to contribute and have their experiential expertise recognised. Meaningful and authentic collaborations between PWLEs and other stakeholders can enhance the quality and relevance of strategies to reduce stigma and discrimination. This is, to our knowledge, the first study of its kind to use a co-production approach to explore experiences and reflections of stigma and discrimination related to mental health from a global perspective. However, the results are not broadly representative of the general PWLE population or suggestive of globally uniform experiences of stigma and discrimination.

**Funding:**

None.

**Research in context**

**Evidence before this study**

We searched PubMed from database inception to January 29, 2024, for articles reflecting co-produced research exploring mental health related stigma and discrimination. We used the broad terms in titles and abstracts (“co-produc∗” OR “co-design∗” OR “participatory”) AND (stigma) AND (mental health), without any language restrictions. Across the articles retrieved through this search we identified limited examples of co-produced research to develop anti-stigma interventions, and other work including, for example, co-produced research to develop mental health services and interventions (without considerations of stigma) and developing strategies to reduce inequalities (with a broad stakeholder group including, but not limited to, people with lived experience of mental health conditions (PWLE)). We did not identify any studies, co-produced with PWLE, that explored stigma and discrimination related to mental health from a global perspective.

**Added value of this study**

This is the first study, to our knowledge, to explore people's experiences and reflections of stigma and discrimination related to mental health from a global perspective, using a co-production approach involving PWLE in all aspects of research conduct. The co-production design and the inclusion of PWLE throughout the research conduct provides a peer-led focus on inquiry and a rich interpretative narrative related to the experience of mental health-related stigma and discrimination, centering voices of PWLE.

**Implications of all the available evidence**

This study bridges the gap of limited meaningful co-produced research in the field of mental health stigma and discrimination, and provides an example of meaningful and authentic involvement and leadership of PWLE in mental health research. It illustrates how collaborations between PWLE and other stakeholders can enhance the quality and relevance of strategies to reduce stigma and discrimination, and provides important insights on key priorities for such work from the perspective of PWLE that need to guide future efforts in this field.

## Introduction

Mental health-related stigma and discrimination are global challenges that require urgent attention.^1^ Stigma refers to the negative attitudes and beliefs that people hold about individuals and/or groups due to their mental health condition, symptoms, or contact with mental health services. Discrimination has been conceptualised as the behavioural component of stigma, act of treating people unfairly based on those same characteristics.^2^ Stigma and discrimination can be experienced in different ways, including perceived (akin to public stigma; awareness of stigmatising negative stereotypes, prejudice, and discrimination endorsed by the general population), endorsed (expressed agreement with negative stereotypes, prejudice, and discrimination), anticipated (expectations of encountering or experiencing stigma and/or discrimination), received (negative interactions and experiences of rejection and devaluation), and enacted (performing behaviours that prejudice or discriminate against the stigmatised person).^3^

Terminology of stigma and discrimination is often used in an interchangeable way, or ‘stigma’ is considered an umbrella term. However, it is important to note the distinction between these concepts, and how a focus on discrimination beyond attitudes and beliefs provides a position of accountability where the argument to eradicate unjust treatment can be backed up with legislation and conventions.^4^^,^^5^

Stigma and discrimination have profound negative impacts on individuals, families, and societies, reflecting poor quality of citizenship and unequal rights. These consequences include exclusion from education, employment, and the community, receiving inferior healthcare compared to people with physical illnesses, loss of property, and reduced chances of getting married.^1^ This prevents individuals from fully participating in society and enjoying their basic human rights, and thus suffer from discrimination.

Efforts to understand the mechanisms and consequences of stigma and discrimination need to center around the perspectives of people affected by these negative impacts. This is right in principle, given the position of ‘nothing about us without us’^6^ and its stance that people with lived experience (PWLEs) of mental health conditions have to hold a central role in the planning of strategies and policies that affect their lives, including conducting research that might inform such strategies. PWLEs can bring unique insights, perspectives and practical solutions through their individual and collective lived experiences, enhancing the validity and relevance of mental health research. This also enables the verification of what is stated in policy and what happens in practice through PWLE experiences and perspectives. Furthermore, PWLE are the key active change agents for stigma reduction and claiming their rights.^1^ This means it is important to not only provide a platform for people to share their experiences of mental health-related stigma and discrimination, but also to explore these topics through research efforts that are led or co-led by PWLEs.

The participation of PWLEs in driving research and policy is limited, especially in relation to healthcare,^7^ although the involvement of PWLEs in research can positively impact interventions and health care services.^8^ For example, a co-produced study found that clients preferred a person-centered model of care over a traditional science-based treatment model.^9^ Similarly, training sessions co-facilitated by PWLEs resulted in decreased stigma among primary care practitioners, with no harm to PWLEs.^10^

This study aims to broaden the perspective of inquiry in research on mental health-related stigma and discrimination through using co-production principles to explore global perspectives and impact of stigma and discrimination. This work enhances the voices of those affected by stigma and discrimination by providing people with lived experience of mental health conditions a platform to share their personal experiences and reflections.

## Methods

### Study design and participants

This study follows co-production principles^11^, ^12^, ^13^ where peer-researchers with lived experience of mental health conditions hold equal space in conceptualising the study, developing research procedures, interpreting results, and writing the study manuscript. It follows the reporting Standards for Reporting Qualitative Research (SRQR)^14^ reporting guidelines. Ethical approval was granted by King's College London's Research Ethics Subcommittee (RESCM-21/22-25892).

Participation inclusion criteria were people (over age 16) identifying as PWLEs who were willing to share their experiences of stigma and discrimination resulting from a diagnosis of a mental health condition, and who had participated in anti-stigma activities. Recruitment followed a self-selecting snowball sampling method.^15^ Potential participants were contacted through mental health organisations collaborating with PWLE (e.g. Global Mental Health Peer Network, Fundación Mundo Bipolar), utilising social media channels and mailing lists to share the survey link. Online informed consent was provided via a mandatory question at the start of the survey, which also indicated that participants had read and understood the information provided regarding the study, including that they met the eligibility requirements to subsequently take part in the survey. No incentive or reimbursement was offered for participation.

Qualitative data were collected via an anonymous online survey (12/01/2021–02/28/2022) available in six languages (Arabic, Chinese, English, French, Russian, Spanish). Translations of the survey description and content were performed by persons speaking the target language and English at native level proficiency, after which the translations were checked by two native speakers. The translation process and reflections on the translations were discussed with the senior author (CS) to ensure equivalence between the original English text and the translation. Open-ended questions explored experiences of stigma and discrimination, experiences regarding diagnoses, language/terminology related to mental health, impact of stigma and discrimination, and involvement with anti-stigma interventions. The questions were developed by a team of people with lived experience, led by author CS, through discussions considering what experiential data on stigma and discrimination would be of value and importance to collect through the survey (e.g. language around mental health conditions, reflections on diagnoses, impact of stigma and discrimination). Proposed questions were discussed further with an international group of experts on stigma and methodologists, including experts by experience of mental health conditions, to consider further topics or dimensions that could be queried (e.g. barriers and facilitators to intervention involvement). Author CS also piloted the questions with members of the Global Mental Health Peer Network (n = 8) and gathered feedback, before the final items for the survey were agreed.

Additionally, socio-demographic information was collected on participants’ age, country of residence, and stakeholder affiliation. The survey questions are provided as an online supplement.

Data were collected from 198 participants, Table 1 outlines respondent characteristics. Data collection was pragmatically determined by the duration of time the online survey was accessible, considered alongside sample size guidance for online qualitative surveys^16^ and principles of data adequacy.^17^ Specifically, when collecting qualitative data via online surveys, samples are usually larger with a reported upper end of around 100 participants or more (to account for how individual responses in datasets generated via online surveys can be brief, and as such larger samples are required to provide richness and depth).^16^ The survey would have been kept open for longer, had data from a sufficiently large number of respondents (n = 100+) not been available at the time of its planned closure. As such, it was considered that these data would be adequate to address the study questions.^17^

**Table 1:** Respondent characteristics (n = 198).

Age, mean (range)	41.3 (20–78)
Stakeholder affiliation^a^	
Person with lived experience of a mental health condition	146
Activist who works or worked, in research, in policy making, and/or has been involved in local community organisations	107
Survey language	
English	61
Chinese	55
Spanish	47
Russian	30
French	3
Arabic	2
Country of residence	
Hong Kong	54
Russia	26
Spain	23
Argentina	21
United States of America	12
United Kingdom	7
Ghana	5
Kenya	4
Other^b^	46

a Respondent could indicate both.

b 3 respondents or fewer: Australia, Belgium, Botswana, Cameroon, Canada, China, Colombia, Denmark, Egypt, France, Georgia, India, Indonesia, Ireland, Kazakhstan, Kyrgyz Republic, Malaysia, Nepal, New Zealand, Nigeria, Norway, Pakistan, Perú, Singapore, South Africa, Switzerland, Syria, Trinidad and Tobago, United Arab Emirates, Uruguay, Uzbekistan, Zimbabwe.

### Data analysis

Responses to question were synthesised through a combination of digital text network analysis using the software Infranodus (version Pro 2022) and thematic content analysis.^18^ Responses were translated to English using Google Translate, with selected accounts cross-checked by native speakers. The English text was uploaded to Infranodus (02/2022) to perform word network analysis. This analysis is based on indicators like word frequency or word clusters (co-occurrence, the frequency in which certain groups of words appear together). Specifically, four team members (PCG, NVSJ, AM, and HL) developed content summaries on data for each survey question, guided by the top eight thematic clusters in the data and the most frequent words/nodes as identified by the software. These results were then used to guide team discussions, where participants’ answers conforming these clusters were explored for a more detailed content review and synthesis of the data. Initial thematic summaries of the survey content were developed based on this, including essential participant accounts to substantiate the results (03/2022). Beginning analysis by digitally identifying key topics present in the data reduces potential researcher bias, while following this structured process with an in-depth manual exploration mitigates limitations of analysis software.^19^ These initial summaries were then refined further by two researchers (PCG, HL), through considering potential latent themes and groupings within these data (07/2022). The emerging results synthesis was reviewed and refined in a workshop with four peer researchers (GM, ML, KV, CS) (09/2022). This involved discussing the initial analysis framework, with peer researchers highlighting particularly salient elements of the emerging results, and whether—given their lived experience perspective—there were any potential omissions in the initial data synthesis (e.g. whether the data included reflections on the importance of peer support and cultural considerations). This discussion guided a further review of the qualitative data to assess whether the topics highlighted by peer researchers were present, and where relevant these elements were integrated to the synthesis. The full author group iteratively reviewed and discussed the themes around which the results were structured, until a final narrative structure was agreed upon (04/2023). This final analytical framework was perceived to appropriately address the aims of the research and accurately and comprehensively reflect the data, accounting for the majority of perspectives.

### Reflexivity statement

Our author team reflects a diverse group of mental health researchers, ranging from pre-doctoral researchers to professors, with and without lived experience of mental health conditions. We represent collaborators from a range of different cultural, geographical, professional, experiential, institutional and disciplinary backgrounds (e.g. psychology, epidemiology, medical sociology, activism, advocacy, journalism). We acknowledge the multiplicity of perspectives, contexts, and influences that inform our contributions to this research, and have employed a reflexive perspective throughout the conduct of this. Our multi-disciplinary approach enhanced the trustworthiness and validity of our data analysis and mitigated potential biases to achieve credibility and rigor of our work.

### Role of the funding source

There was no direct funding source for this study.

Authors PCG, HL, NVSJ and AM had access to the dataset, and all authors (CS, SK, ML, HL, AM, GM, KV, NVSJ, PCG) had joint final responsibility for the decision to submit the work for publication.

## Results

The results reflected five themes: 1) the role of language and words; 2) the role of media in perpetuating and reducing stigma; 3) societal reactions to mental health conditions and strategies to cope with these; 4) knowledge about activities to reduce stigma and discrimination and their impact; and 5) personal involvement in activities to reduce stigma and discrimination. These themes are presented next, alongside select supportive participants’ accounts (additional quotes in online supplement).

### The role of language and words

Language was considered a vehicle that shapes attitudes, and contains values, emotions, and perceptions of mental health conditions. Respondents were aware of and/or had directly experienced multiple negative terms and derogatory remarks about mental health and themselves.

- Being addressed ‘psycho’ or ‘mental’ whenever we even try to articulate our mental health struggle and overthinking we experience, [can] make us feel unsafe to share it [mental distress] … fear of judgement make it hard and unsafe for us to admit we have mental health issues

(Participant from Russia)

Participants spoke of both unacceptable and suitable terms, and they recognised that appropriate language and words were highly context dependent.

- Some terms are insulting to persons with lived experience - although acceptable terms are very culturally and context specific

(Participant from Denmark)

- ‘Psychosocial disability’ and ‘crazy’ depends on the tone and meaning with which it is used

(Participant from Argentina)

Another point raised was the language used by health professionals, and the potential to change discourse in mental health care. Sensitive language was important, particularly during consultations. PWLEs emphasised that their views need to be included when selecting appropriate terminology, reflecting person-centred, recovery-focused language and neutralising words, rather than focusing solely on the mental health condition.

- Persons living with the conditions must be consulted on words they would prefer to be addressed with.

(Participant from Ghana)

Another aspect was language in diagnostic classifications. Participants expressed mixed views on the usefulness of these systems. Their perceived benefit was related to diagnosing, offering treatment for mental health conditions, and supporting communication in education and research. However, diagnoses could also contribute to stigma, discrimination and labelling, and be too medical, ignoring the social context of PWLEs.

- I think that such systems can be helpful by the means of establishing a lexicon of existing conditions. However, they should be used in conjunction with a person-centric approach for diagnosing mental health conditions. Ignoring the diversity of human experience that often leads to mental health conditions would lead to improper diagnosis, and therefore, treatment.

(Participant from India)

Language was mentioned also in relation to media; this is expanded in the next theme.

### The role of media in perpetuating and reducing stigma and discrimination

Respondents discussed how media, with its language and imagery, could influence general attitudes and culture with the potential for both negative and positive impacts.

Negative effects included using inappropriate language, reinforcing stereotypes (e.g. parodying people with mental health conditions or portraying them as dangerous), bullying and trolling, and sharing misinformation.

- For example, a programme talking about anorexia on the television, while it was great to expose the difficulties of people, it gave a lot of mis-information and did not give the complexities and realities of the illness. This could, while trying to reduce stigma, actually enhance it.

(Participant from England)

Media could also have a positive impact on stigma and discrimination with appropriate policies and guidance. Person-centred, balanced, and realistic/factual media content on mental health conditions could normalise the topic. For example, the media could play a helpful role in education about mental health. Furthermore, social media could reach those who might not consume traditional media. Respondents called for strategies on how to use appropriate language in the media, protecting people who speak about their mental health, keeping a balanced approach to inform about mental health, educating and raising awareness, providing a platform for PWLEs to share their stories, and building communities for people with mental health problems to share and foster hope.

- Promoting information about positive initiatives, anti-stigma events, peer-support groups, inviting persons with lived experience to speak about recovery and maybe other topics too, just to show we are people outside of the walls of institutions and not real serial killers.

(Participant from Georgia)

### Societal reactions to mental health conditions and strategies to cope with these

Societies were considered unsympathetic, unwilling to understand or listen, with many people having limited knowledge about mental health conditions.

- It is difficult to make others understand that it is not our fault [that we have a mental illness] or that of others.

(Participant from Switzerland)

Media, culture, and traditional beliefs were seen as contributing to societal reactions and misunderstandings. Many respondents expressed concerns and difficulties around anticipated or experienced consequences of disclosing their mental health conditions. They feared changes in relationships with friends and family (e.g. not taking the person's mental health condition seriously, believing negative stereotypes). Some feared abandonment, isolation, and estrangement. Negative reactions were also expected in relation to disclosing mental health conditions at work and during interactions with healthcare professionals.

- But the most stigma I have faced was by health professionals who have no understanding of mental illness. They seem unable to comprehend that people facing mental illness are still people just like anyone else.

(Participant from Singapore)

In terms of strategies to cope with negative reactions, respondents suggested that receiving and providing support, working with mental health professionals, and learning more about the nature of mental health is helpful. Their preferences varied: some mentioned that not disclosing mental health conditions and trying to adjust to social norms helped, while others tackled negative societal reactions through talking openly about mental health and raising awareness. Important resources to cope with negative reactions were empathic relations and support systems (family, friends, co-workers, mental health organisations, peers). Also, supportive mental health professionals were crucial, albeit not always available.

- My therapy and talking to people who understand me because the same thing has happened to me or because they are mental health professionals and understand the subject.

(Participant from Argentina)

For some an important coping strategy was to engage in advocacy and anti-stigma activities, expanded in the theme ‘Personal involvement in activities to reduce stigma and discrimination’.

### Knowledge about activities to reduce stigma and discrimination and their impact

According to the respondents, changes in stigma and discrimination could be achieved through awareness-raising and education initiatives for key target groups (e.g. public, students, healthcare practitioners, families) and engaging with media. Respondents were aware of such activities and mentioned courses and campaigns to raise awareness about mental health, using appropriate language, and promoting equal opportunities. These strategies were delivered on social and mass media, and were sometimes promoted at specific dates/events (e.g. World Mental Health Day).

- Informative activities on mental health and various mental disorders in parks, schools, universities, and cultural forums. Online visual materials. Individual efforts of mental health professionals when making lectures or publishing visual or written materials on the Internet.

(Participant from Egypt)

Structural-level efforts were also mentioned. Examples included lobbying for changes in Mental Health Acts, National Disability Commissions, legislations, and policies. Updating laws in line with international human rights standards was suggested as a key step in addressing stigma and discrimination. However, sometimes these acts and policies were mainly considered general guiding principles, as there were no appropriate resources to enact them. Overall, despite the many reported anti-stigma initiatives, a need for more activities, publicity, and advocacy was identified. A focus on equality was essential, specifically in employment settings.

In terms of positive impacts of anti-stigma activities, respondents had noticed improvements through personal feedback, surveys, improved social relationships and other tangible changes in the community or community-based organisations (e.g. improved social interactions, open conversations, access to employment, improved social support, and a sense that the next generation was more accepting).

- There has been increased media coverage on mental health and a general sense of tolerance has grown.

(Participant from Kenya)

Participants felt that anti-stigma activities were not truly effective if their impacts remained at an individual level. Importantly, structural-level change through legislation was considered key evidence of stigma and discrimination decreasing.

- The way I see it [anti-stigma activities], it's not effective because the government has not done anything to reduce the stigma for people with psychosocial disabilities

(Participant from Indonesia)

Another factor reported as a characteristic of a weak campaign was if PWLEs were involved only in a superficial or tokenistic manner: this is expanded in the next theme.

### Personal involvement in activities to reduce stigma and discrimination

Respondents have participated in anti-stigma activities with many different target groups, including schools, universities, workplaces, church gatherings, hospitals, political stakeholders, community leaders and healers. These initiatives had involved sharing personal narratives in person and online, individually or through wider campaigns/programmes. Some activities had incorporated creative means (e.g. films) and developing courses and training programmes (e.g. mental health awareness).

- Before getting sick with refractory Schizophrenia, I did volunteer work at a home for the elderly mental ill. I also speak openly on the web, YouTube, Instagram, Twitter, etc. about Schizophrenia. I make it open. I cut the ice.

(Participant from Spain)

Challenges with anti-stigma campaign involvement included negative reactions from others, the burden of reliving past experiences, encountering upset event attendees, difficult interpersonal encounters due to increased visibility of lived experience, and sensing that one's involvement was only tokenistic. Also, practical challenges were mentioned by respondents (e.g. remembering what to say, scheduling of event, problems with effectively engaging with target audiences).

- [It is] hardest to share a story or deliver a workshop if a safe space is not created first.

(Participant from Norway)

Factors facilitating involvement included training and support enabling PWLEs speaking out and sharing their narratives safely, practical organisational support, peer support, payment/compensation for their time, skills, and expertise, feedback on programme impacts, and a personal sense of satisfaction. Sharing experiences and engaging with peers was considered particularly important, as it facilitated connections, shared understandings of experiences, enjoyment of equal status, and a sense of safety, empathy, and support.

- It takes training to turn one's own experience into a story of hope.

(Participant from Hong Kong)

- It's the confidence in my skills, and the fact that I'm treated like a teacher. Like another.

(Participant from Switzerland)

- I also treasure the peer support within the organization. We need a safe organization to grow and learn.

(Participant from Hong Kong)

Meaningful involvement (ideally leadership) of PWLE was called for throughout anti-stigma programme planning, delivery, and evaluation. Taking part could be supported through providing training on how best to share one's story of living with a mental illness, payment for the expertise and contributions, and involving people at an appropriate stage in their recovery.

- Include them [PWLEs] from the beginning of the process in the reflection on the program (not just like “testimony machines”).

(Participant from Switzerland)

- Be convinced that people with lived experience in Mental Health are essential for the execution of programs.

(Participant from Spain)

Personal impacts of participating in anti-stigma activities were perceived as valuable. Although opening up could be difficult initially, candidness and meaningful conversations had led to improved self-confidence, self-compassion, agency, self-worth, acceptance, inner strength and reduced self-stigma.

- [taking part in anti-stigma activities] helped me feel like a useful person again.

(Participant from Spain)

## Discussion

This study used a co-production approach to explore experiences and reflections of stigma and discrimination related to mental health from a global perspective. The results of this study highlight that people with mental health conditions are aware of and experience stigma and discrimination across core domains of daily life. A further key finding was the importance of recognising the central role PWLE can play in efforts to reduce stigma and discrimination.

Participants’ accounts outlined how experiences of stigma and discrimination often manifested through inappropriate language and words in interpersonal exchanges—both in informal settings as well as with healthcare providers—including terminology related to diagnostic classification. They reported that terms used for mental health conditions were often unacceptable, derogatory, or insensitive. Although emphasis was placed on the importance of using appropriate language, contextual specifics can mean that identifying a set of universally accepted terms is unlikely.^20^^,^^21^ The principle of using person-first language^22^ is important to avoid defining a person as a diagnosis and losing the recognition of them as an equal human being with respect to human rights regardless of their mental health status.

Media was highlighted as a key mechanism to influence stigma and discrimination. For decades this powerful institution has contributed to spreading misinformation, inappropriate language and reinforcing negative stereotypes about mental illness. Previous studies have likewise emphasised how media reporting reflects public opinion and stigma.^23^^,^^24^ Conversely, media also has a central role and responsibility in tackling stigma and discrimination.^25^^,^^26^ Proactive protection of the integrity and dignity of people and sensitivity about personal experiences are core factors by which media can decrease sensationalism, stereotypical portrayals of mental health, and public trolling/bullying. PWLEs are uniquely positioned to strategically assist in the development of policies and advise on how to create neutral media content and information that is not stigmatising, discriminatory or posing risks of human rights violations.

Media can also provide a platform for public figures and celebrities to speak out about mental health, which can help to reduce stigma.^27^ Such disclosures can normalise mental health conditions and destigmatise help-seeking, however this approach may mask the discrepancy of how public figures’ social context and access to resources and support is very different than for the general population. Providing opportunities for sharing and understanding accounts of living with a mental health condition by non-public figures could redress this imbalance and ensure that a broader range of voices and perspectives are shared.

An important theme in our findings was that PWLEs need to be provided opportunities for meaningful involvement in efforts to reduce stigma and discrimination. The role of PWLEs in anti-stigma activities has to be appropriately supported, recognising the value of experiential expertise. Participation needs to extend beyond a tokenistic role to genuine collaboration, reflecting active contributions throughout the planning, delivery, and evaluation of anti-stigma activities, in either programme lead or co–lead capacity. This is in line with initiatives that have been implemented in past,^28^^,^^29^ and reinforces one of the key messages of the Lancet Commission on Ending Stigma and Discrimination in Mental Health.^1^ By acknowledging and confronting stigma and discrimination people can reclaim their sense of dignity and self-worth, connect with others who share their experiences, and advocate for their rights and needs. Fundamentally, the involvement of PWLE in stigma-reduction activities adheres to the core principle of ‘nothing about us without us’; a motto promoting the principle of participation and equalisation of opportunities for people with disabilities, contributing to the development of truly inclusive societies.^6^

A further consideration for efforts to reduce mental health stigma and discrimination, in addition to PWLE involvement, is to link the concept of discrimination more strongly to negative consequences of stigma, such as the loss of opportunities and rights. Discrimination is a transgression of fundamental local and global laws and human rights, and for example the Convention on the Rights of Persons with Disabilities is an international treaty of the United Nations to protect persons’ rights and dignity.^5^ Recognising discrimination and considering a human rights perspective in efforts to reduce stigma could increase accountability and drive positive action. It could be interesting to explore whether there are differences in experiences of stigma and discrimination, and differential preferences in involvement in anti-stigma activities and other work that promotes rights and inclusion, and how such involvement could be best achieved, between people with different types of lived experience of mental health conditions or those who consider themselves to be activists in the area versus those who do not. Additionally, future studies could explore differences in experiences of stigma and discrimination within and between different global regions, to highlight and learn how experiences might differ depending on contextual differences in relation to cultural influences, health systems resources, religion et cetera.^30^, ^31^, ^32^ The nature of the data collected for this study did not allow for this level of nuance to be explored in the current analyses.

Strengths of the work include its co-production approach, involving PWLE in all aspects of research conduct. In particular, the data analysis was strengthened by involving researchers with and without lived experience of mental health conditions. This is, to our knowledge, the first study of its kind to use a co-production approach to explore experiences and reflections of stigma and discrimination related to mental health from a global perspective, supporting participation through a survey available in multiple world languages and reaching participants in over 30 countries across the world. The results provide a useful direction for future research in the field, by using comparable methods of enquiry and setting meaningful research questions for further exploration.

The results build on a global sample of PWLEs, however, it is not broadly representative of the general PWLE population or suggestive of globally uniform experiences of stigma and discrimination. For example, as all respondents had taken part in anti-stigma activities, these experiences may have influenced their perspective on stigma and discrimination, or perhaps specific experiences of negative social consequences and interactions had led to their involvement with anti-stigma activities in the first place. It might also be that experiences of stigma and discrimination vary depending the nature of a person's mental health condition or diagnosis; a dimension we did not focus on in this study. We also did not collect information that would have allowed for considering the results by gender or other subgroups, as this level of detail was beyond the scope of the current study. This study was unable to ascertain whether respondents had experienced mental health challenges beyond participants' self-identification, as the data were collected via an anonymous online survey. Our stance is, however, that no qualifier is required beyond a person considering themselves a person with lived experience. It is, however, recognised that this sampling strategy might have introduced a level of self-selection bias influencing sample representativeness. Although the qualitative survey questions were designed to elicit rich, open-ended responses, interview-based data collection would likely have yielded more in-depth insights.^16^ The online survey approach facilitated data collection from a larger number of participants that what would have been feasible via interviews. It is possible that the snowball sampling led to a sample overrepresenting people involved with the mental health organisations through which the survey link was initially shared. However, this approach reached many PWLEs active in stigma-reduction initiatives and helped to broaden the sample by sharing the link through multiple organisations and on social media and via email lists. Translating responses via Google Translate might have introduced errors, but any potential issues were flagged up and selected accounts were cross-checked for quality by native speakers.

This study centered voices of people living with mental health conditions through sharing experiences and reflections of stigma and discrimination. Eradicating these negative experiences relies on collective action by multiple stakeholders (international agencies, governments, policymakers, healthcare sector, research institutions, mental health charities, media, communities, families and PWLE), and aligning laws and policies with human rights treaty to accomplish effective accountability within policy and practice. The findings position PWLEs as key agents for change who need to be consistently supported to lead or co–lead activities to reduce stigma and discrimination. Whilst there is a growing global recognition of the critical role of PWLEs as key partners in such efforts, it is essential to strengthen their effective engagement and inclusion through principles such as empowerment, valued contributions, equal power balance, and non-tokenism. Meaningful and authentic collaborations between PWLEs and other stakeholders can enhance the quality and relevance of strategies to reduce stigma and discrimination. This study is an important example of how key priorities for such work from the perspective of PWLEs can guide efforts in this field.

## Contributors

The study was conceived by CS and PCG. CS, PCG and HL developed the survey. Data were analysed by HL, NVSJ, AM, PCG, GMC, ML, KV, and CS. PCG and CS structured the manuscript, with all authors (CS, SK, ML, HL, AM, GMC, KV, NVSJ, PCG) contributing to subsequent drafting and PCG refining the combined final text. Authors PCG, HL, NVSJ and AM had access to and have verified the underlying dataset. All authors approved the final manuscript and had final responsibility for the decision to submit for publication.

## Data sharing statement

Raw qualitative data cannot be shared due to ethical restrictions. Deidentified qualitative data can be shared on reasonable request to the corresponding author providing appropriate ethical approvals are granted.

## Declaration of interests

All authors declare no competing interests.
