# Supplementary material for: Exploring perspectives of stigma and discrimination among people with lived experience of mental health conditions: a co-produced qualitative study
Source: eClinicalMedicine. 2024 Feb 28;70:102509. doi: 10.1016/j.eclinm.2024.102509 (PMC10912051; doi:10.1016/j.eclinm.2024.102509)
Supplement: Supplementary file [file mmc1.pdf]

Supplementary material: Survey Instrument and Additional Participant Quotes

Contents

1. Survey Instrument 1

2. Additional Participant Quotes 4

---

1. Survey Instrument

Global survey for people with lived experience of mental health conditions

Welcome to Our Survey

We are asking for your perspectives on stigma and discrimination in mental health based on your lived experience of a mental health condition.

This study aims to collect the lived experiences of people with mental disorder regarding stigma and discrimination from across continents. The data collection takes place in the context of preparing the Lancet Commission on Stigma and Discrimination in Mental Health (LCS) report. The Lancet is a renowned medical journal, at the Lancet Commissions are reports on urgent and often neglected or understudied health issues.

The LCS report will summarise the current state and provide recommendations on how to reduce stigma related to mental health conditions. In this context, the lived experiences of people with mental health conditions will be collected through this online survey.

This survey consist of questions, in which you are asked to describe your experiences regarding diagnoses, language / terminology related to mental health, impact of stigma and discrimination on your life, experiences with anti-stigma interventions, and your opinion of how digital and traditional media contribute to stigma and discrimination. The information from this survey will be used to highlight the importance of reducing stigma and discrimination related to mental disorders worldwide.

Completing this questionnaire should take approximately 15-20 minutes.

You can find further information regarding the study here: https://indigo-group.org/the-lancet-commission-on-stigma-and-discrimination-in-mental-health-lcs/

You can download the survey information sheet here.

By confirming that you have read and understood the study information you indicate your consent to take part in this study. Your participation is fully voluntary and anonymous.

If you have any questions please contact [details redacted].

---

* 1. I have read and understood the information provided and I agree to take part in this research project.

  o Yes

2. What is your age?

3. In what country do you live?

* 4. I am a ... (tick all that applies)

- person with lived experience of a mental health condition
- an activist who works or worked, in research, in policy making, and/or have been involved in local community organisations/NGOs

5. Have you ever participated in activities / meetings / projects to reduce stigma and discrimination related to mental health conditions?

- Yes
- No

6. Please rate the following statements according to what best fits your views

All questions rated as:

- *Strongly Disagree*
- *Disagree*
- *Neither Agree or Disagree*
- *Agree*
- *Strongly Agree*

Stigma and discrimination do negatively affect most people with mental health conditions

Stigma and discrimination can be worse than the impact of the mental health condition itself

Overall stigma and discrimination in my country have been reducing over the last decade

People with lived experience of mental health conditions must lead anti-stigma efforts

My government should invest in a long-term national programme to reduce stigma and discrimination

People with lived experience of mental health conditions should be treated as well as people with physical health conditions

The media is a major factor in making stigma and discrimination worse

The media could play a major role in reducing stigma and discrimination

[Open-ended questions considered for this publication start here]

LANGUAGE, WORDS AND TERMS

7. How does it matter to you what language / words / terms are used when talking about mental health conditions? How does language matter?

8. Are there terms or words which you think are inappropriate to be used? Please give examples (e.g. crazy, mad, challenged)

9. What suggestions/examples do you have for less stigmatizing words / terms?

10. How do you think we need to go forward in the future in relation to words /terms when talking about mental health conditions, diagnoses, or treatments?

11. Do you believe that diagnostic classification systems (e.g. ICD or DSM*) are useful or do they contribute to stigma and discrimination? Why?
*(*Guideline which is used by mental health professionals to diagnose a person based on their symptoms)*

IMPACT OF STIGMA

12. Leaving aside your own mental health problems and focusing on the wider societal reactions (e.g. from family, neighbours, friends, colleagues, or other societal groups) in relation to your mental health condition, what has been the most difficult aspect for you?

13. What has helped you to cope with negative societal reactions towards your mental health problems?

ANTI-STIGMA PROGRAMS
**If you don't have knowledge or experience of any of questions 13 to 19, you can move onto question 20.*
14. Can you provide specific examples how you participated in activities / meetings / projects (e.g. activities in schools, at work, with health staff, the police) to reduce stigma and discrimination related to mental health conditions? *(If you don't have knowledge or experience of this, you can move onto the next question)*

15. How do you know whether the above mentioned activities have been effective or not within your clinic, hospital/primary care or government level?

16. What were your experiences of such activities? How did these help you or not?

17. What kind of activities in your region or country do you know about for reducing stigma and discrimination?

18. If you have been included in delivering anti-stigma programs (e.g. workshops, lectures), can you tell me/provide examples, which has been the most difficult for you?

19. If you were ever included in delivering anti-stigma programs (workshops, lectures, etc.), what factors made your experience easier or rewarding (e.g. help from the organization, training, payment, peer support, or supervision)?

20. What are your recommendations to providers of anti-stigma programs how best to include people with lived experience in the program delivery? Do you have specific examples that worked well?

STIGMA IN THE MEDIA
**If you don't have knowledge or experience of questions 21 and 22, you can move onto question 23.*
21. What kind of activities (e.g. political changes, new laws) in your region or country do you know about for reducing stigma and discrimination?

22. How do you know whether stigma reduction activities/initiatives have been effective or not?

23. In your view, how do you think various types of digital media (e.g Facebook, Instagram, TikTok, Weibo, WhatsApp, Twitter) or traditional media (newspapers, TV, radio) can sustain or increase stigma and discrimination related to mental health conditions?

24. In your view, how do you think various types of digital media (Facebook, Instagram, TikTok, Weibo, WhatsApp, twitter, etc.) or traditional media (newspapers, TV, radio) can reduce stigma and discrimination related to mental health conditions?

OVERALL COMMENTS

25. Please briefly add any other comments you would like to tell me about the most important issues in reducing stigma and discrimination, which I have not included in the above questions and are important to you.

Thank you for your participation

2. Additional Participant Quotes

**1. The role of language and words**
**On negative terms and derogatory remarks**
Being addressed 'psycho' or 'mental' whenever we even try to articulate our mental health struggle and overthinking we experience, make us feel unsafe to share it [mental distress]… fear of judgement make it hard and unsafe for us to admit we have mental health issues (Participant from Russia)
They are inappropriate words of crazy, insane and retarded. (Participant from Argentina)
loony, wacko, windae licker, rocket....... (Participant from Scotland)
sick, monster, abnormal (Participant from China)
Disorder of… schizo, autism, bipolar, phobia that we find all over the place in the media. Paradoxically I prefer the sympathetic words of crazy, deranged, crazy, zinzin… (Participant from Switzerland)
schizophrenic, wacko, psychotic, queer, troll, retarded, mogul (Participant from Argentina)
**On acceptable language**
Instead of bipolar disorder it should be called the disease of emotions. The word bipolar has a lot of stigma and has been popularized in movies and TV for the worse. (Participant from Spain)
Use the terminology that is appropriate for the experience and just don't use them as insults (Participant from South Korea)
**Appropriateness of language is context-dependent**
It matters in the context that it was used and the semantics of the words and the whole sentence if used together. (Participant from Malaysia)
It is critical. Language has context and connections. (Participant from Australia)
Context and/or intent matters. If language is being used to discredit or de-legitimise an individual living with a mental illness, it is stigmatizing. (Participant from United Kingdom)
**Language used by health professionals**
I think neutral, person-first language, and empowering languages are helpful. E.g. people with mental illness. I think Recovery vocabulary are helpful to replace terms that are heavily medical/professional oriented. (Participant from Hong Kong)
Training people to use non-intrusive, word, e.g. using proper medical terms, will cause less trauma. (Participant from South Africa)
Only kind words and descriptions. (Participant form Zimbabwe)
**Views on diagnostic classifications**
Useful, but not conducive, as long as it is used correctly and makes the public have a better understanding of the disease. (Participant from Hong Kong)
Not useful -these are a guideline; each person's lived experience is completely different. (Participant from South Korea)
**2. The role of media in perpetuating and reducing stigma and discrimination**
Facebook groups, forums, instagram accounts, online communities can all help to find others who have struggled with similar issues and feel less alone. Simultaneously they expose these issues to a broader public and educate them through first-person narratives which are easier to empathise with than traditional media, yet anonymous so they can protect the posters. (Participant from Singapore)
For example, there has been a change in the language used by media while reporting suicide in India. From committed suicide, the language now used is died by suicide. This subtle change in language takes away from the idea that suicide is a crime. (Participant from India)
3. **Societal reactions to a mental health problem and strategies to cope with them**
**Experiences of negative reactions**
Trying to navigate work expectations and familial expectations of how I should function as someone in my age group, when sometimes functioning the way, they expect me to is almost impossible (Participant from South Korea)
**Approaches to coping**

Hide it (Participant from Spain)
Finding kind people to talk to. Accepting who I am for myself and accepting other people's difficulties in accepting me. Learning to let go. (Participant from England)
**4. Knowledge about activities to reduce stigma and discrimination and their impact**
**Knowledge of activities**
For Kenya we have a mental health day, mental health month and NGO, CBOS, FBO that openly seek to reduce stigma within the general public (Participant from Kenya)
**Assessing their impact**
Personal feedback from hundreds of people who have been positively impacted (Participant from UK)
**5. Personal involvement in activities to reduce stigma and discrimination**
**On participation**
Organized a youth exchange on the topic of raising awareness of the problem (Participant from Russia)
**Challenges in taking part**
I think it's to share past experiences that might bring back some unpleasant memories! (Participant from Hong Kong)
Telling my story and the emotions it invokes in others (Participant from USA)
**Factors facilitating involvement**
Critical to pay people if possible. Sharing a lived experience is a vulnerable thing, and a skill. It should be paid in the same way other work should be paid. (Participant from Norway)
Personal experience and the presence in the team of people who also had such experience (Participant from Russia)
**Value of peer-support and peer-led activities**
Being supported by people who understand (Participant from Australia)
Only someone with lived experience can appreciate the shame that often comes with mental illness and only a person with lived experience can share how they faced it. (Participant from USA)
I also treasure the peer support within the organization. We need a safe organization to grow and learn. (Participant from Hong Kong)
Let experienced people choose the safest way to share with them, colleagues create a safe environment, and provide support (Participant from Hong Kong)
**Recommendations for future work**
Provide training and workshops to make it easier for me to reflect such as searching for my soul (Participant from Hong Kong)
For me they were very enriching, because with each job I did I learned a little more about the problem and acquired a more critical point of view about the treatment of the media and about stigma in general. (Participant from Spain)
**Impact of taking part**
Some of the activities were fun, relaxing and helpful in the short-term (eg. on the same day), but often they were surface-level because they did not delve deep into the topic of mental illness and if they did, only focused on easily understandable disorders such as mild anxiety. The more helpful activities were sometimes unrelated, but involved deep conversation and community building - eg. dialogues among sexual assault survivors that touched on topics of trauma and mental illness (Participant from Singapore)
